# DeepFHR: intelligent prediction of fetal Acidemia using fetal heart rate signals based on convolutional neural network

**DOI:** 10.1186/s12911-019-1007-5

**Published:** 2019-12-30

**Authors:** Zhidong Zhao, Yanjun Deng, Yang Zhang, Yefei Zhang, Xiaohong Zhang, Lihuan Shao

**Affiliations:** 1 College of Electronics and Information, Hangzhou Dianzi University, Hangzhou, China; 2 Hangdian Smart City Research Center of Zhejiang Province, Hangzhou Dianzi University, Hangzhou, China; 3 School of Communication Engineering, Hangzhou Dianzi University, Hangzhou, China

**Keywords:** Fetal acidemia, Computer aided diagnosis system, Continuous wavelet transform, Convolutional neural network, Fetal heart rate

## Abstract

**Background:**

Fetal heart rate (FHR) monitoring is a screening tool used by obstetricians to evaluate the fetal state. Because of the complexity and non-linearity, a visual interpretation of FHR signals using common guidelines usually results in significant subjective inter-observer and intra-observer variability. Objective: Therefore, computer aided diagnosis (CAD) systems based on advanced artificial intelligence (AI) technology have recently been developed to assist obstetricians in making objective medical decisions.

**Methods:**

In this work, we present an 8-layer deep convolutional neural network (CNN) framework to automatically predict fetal acidemia. After signal preprocessing, the input 2-dimensional (2D) images are obtained using the continuous wavelet transform (CWT), which provides a better way to observe and capture the hidden characteristic information of the FHR signals in both the time and frequency domains. Unlike the conventional machine learning (ML) approaches, this work does not require the execution of complex feature engineering, i.e., feature extraction and selection. In fact, 2D CNN model can self-learn useful features from the input data with the prerequisite of not losing informative features, representing the tremendous advantage of deep learning (DL) over ML.

**Results:**

Based on the test open-access database (CTU-UHB), after comprehensive experimentation, we achieved better classification performance using the optimal CNN configuration compared to other state-of-the-art methods: the averaged ten-fold cross-validation of the accuracy, sensitivity, specificity, quality index defined as the geometric mean of the sensitivity and specificity, and the area under the curve yielded results of 98.34, 98.22, 94.87, 96.53 and 97.82%, respectively

**Conclusions:**

Once the proposed CNN model is successfully trained, the corresponding CAD system can be served as an effective tool to predict fetal asphyxia objectively and accurately.

## Background

Fetal distress caused by hypoxia can lead to various abnormalities that can be divided into life-threatening and non-life-threatening events during the process of childbirth. Since the brain of a neonate is easily influenced by oxygen supply, a lack of oxygen can cause serious damage to the brain and even death [1]. Hence, to detect fetal acidemia early, we need a powerful technique that can monitor the fetal state in real time, and once an abnormal situation occurs, alert obstetricians to intervene in a timely manner before there is permanent damage to the fetus.

In clinical practice, cardiotocography (CTG) involves the continuous recording of the fetal heart rate (FHR) and uterine contraction (UC) signals and is routinely adopted by doctors to monitor and assess the fetal state during pregnancy and delivery [2, 3]. Unfortunately, due to the complexity of fetal physiological dynamics, which are regulated by neurological feedback loops, the visual analysis of FHR signals using common guidelines usually leads to high intra-observer and inter-observer disagreement among experts [4, 5]. In practice, obstetricians perform multiple subjective evaluations and thereby minimize diagnostic error. However, the main issue of the aforementioned process is the inability to be quantitatively realized, and obstetricians make decisions based on their individual experience [6, 7]. Consequently, the incidence rate of unnecessary cesarean sections (CSs) caused by subjective error is increasing and has become the main driving force in the search for a more objective analysis of the FHR signal [8].

In recent decades, to overcome the inherent defects of visual interpretation of FHR signals, many researchers have attempted to design reliable computer-aided diagnosis (CAD) systems consisting of automatic signal processing and evaluation [9]. Many advanced developments in the biomedical engineering field have been extensively used in FHR signals, such as frequency domain analysis [10], nonlinear features (entropy, complexity, etc.) arising from the domain of adult heart rate variability (HRV) analysis [11, 12], and others [13].

Furthermore, over the past several years, the existing CAD systems have been implemented with the application of machine learning (ML) algorithms to automatically classify pathological fetal events from normal events. Table 7 summarizes the related state-of-the-art work focusing on the above aim. Notably, earlier efforts on FHR-based CAD systems employed the conventional ML approaches and followed the same procedure: (i.) signal preprocessing (i.e., denoising), (ii.) feature extraction, (iii.) feature selection, and (iv.) final classification. These methods based on predictive learning classifiers mostly relied on complex hand-crafted features. For example, Czabanski et al. [14] designed an expert system to predict neonatal acidemia using a two-stage analysis based on weighted fuzzy scoring (WFS) and least square support vector machine (LS-SVM) and obtained performance with an accuracy (Acc) and quality index (QI) of 92.0 and 88.0%, respectively. Fanelli et al. [15] introduced a new nonlinear parameter based on the phase-rectified signal average (PRSA) for the quantitative assessment of fetal well-being and achieved an area under the curve (AUC) of 75% using the univariate analysis method. Comert et al. [16] applied an artificial neural network (ANN) and performed a classification with an Acc, sensitivity (Se), and specificity (Sp) of 92.40, 95.89 and 74.75%, respectively. Obviously, the feature engineering has dominated over conventional methods involving the difficult process of informative feature extraction and optimal feature selection, which is time-consuming, and may result in loss of physiological information regarding the fetus during the overall procedure.

Traditional ML methods usually exist the concept of the “black box“where even their designers cannot provide explanations/justifications explain why the artificial intelligence (AI) can accomplish the specific decision. Holzinger focused on the explainable AI, which made more re-traceable, explainable and reliable decisions [17].

In this study, we propose a deep convolutional neural network (CNN) framework aimed at FHR classification. Compared to the previously mentioned traditional methods, the CNN-based approach is completely data-driven and does not need to explicitly define the essential steps, namely, feature extraction and selection and classification [18]. Actually, these steps are all incorporated into the CNN model by means of self-learning informative features from the input data. CNNs have already yielded great achievements in image classification since they consider the spatial structure of the input data and avoid the curse of dimensionality [19]. Due to the attractive advantages, CNNs are extensively utilized in the medical field for the purpose of designing screening tools that automatically assist clinicians. For example, Acharya et al. designed the CNN structure to diagnosis coronary artery disease using an electrocardiogram (ECG) signal and achieved high accuracy of 95.11% [20]. In addition, Li et al. applied the 1-dimensional (1D) CNN to classify FHR signals and obtained the Acc of 93.24% [21]. Additionally, Comert et al. also proposed a novel approach to detect fetal hypoxia based on a deep CNN with transfer learning using the FHR signal and short term Fourier transform (STFT) [22].

**Fig. 1:**
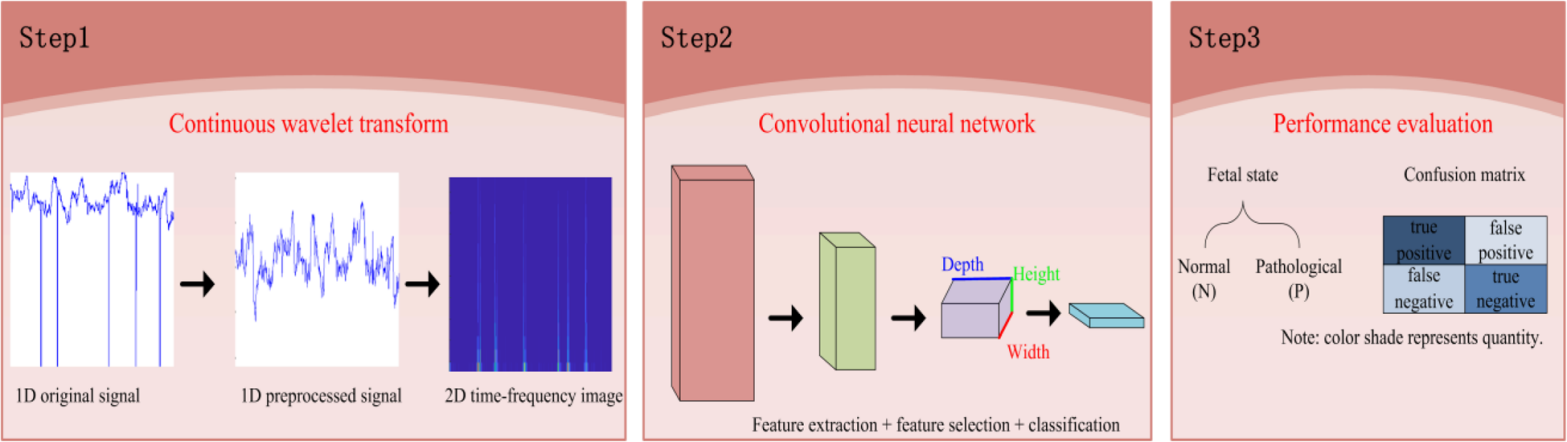
An overview of our proposed CAD system for intelligent prediction of fetal acidemia

Notably, a traditional CNN model requires 2D images as input, but most biomedical signals only have a 1D structure. Therefore, after signal preprocessing, we apply the continuous wavelet transform (CWT) to pure FHR signals and obtain 2D time-frequency images, which can reflect the local hidden characteristic information of the FHR signals in both the time and frequency domains [23]. We evaluate our proposed algorithm on the freely open-access database, which is available from physionet.org [24, 25]. Figure 1 shows the entire framework proposed in this work.

In summary, automatic processing and further classification of FHR signals are indispensable components of CAD systems that satisfy the primary goal of this study, which is to facilitate the intense work of obstetricians and to assist them in making appropriate medical decisions to better protect the health of pregnant women and fetuses. The remainder of the paper is organized as follows: Section 2 introduces the database and gives a detailed description of the overall methodology of our proposed system; Section 3 depicts the corresponding experimental results and presents the discussion, including a comparative analysis with existing approaches; and Section 4 concludes the entire work and proposes directions for future work.

## Methods

### Database description

The data used in this work originated from CTU-UHB, a freely open-access database of a subset with 552 intrapartum CTG recordings that were acquired between 2009 and 2012 in the obstetrics ward of the University Hospital in Brno, Czech Republic [25]. Though these three sets of 102, 412 and 35 records were acquired by means of a scalp electrode, ultrasound probe and both techniques, respectively, expert evaluation of the CTG data based on annotation of the signals were made by 9 expert obstetricians (following FIGO guidelines used in the Czech Republic) including heterogeneous/confidence for each signal. All FHR traces were sampled at 4 Hz [46]. The main parameters and their respective distributions are depicted in Table 1.

**Table 1:** An overview of the available information in the open access CTU-UHB CTG database

Information	Mean	Min	Max
Maternal age (MA, year)	29.6	18	46
Gestational age (GA, week)	40.0	37	43
pH	7.23	6.85	7.47
Base deficit in extracelluar fluid (BDecf, mmol/L)	4.60	−3.40	26.11
pCO2	7.07	0.70	12.30
Base excess (BE)	−6.38	−26.80	−0.20
Apgar 1 min	8.3	1	10
Apgar 5 min	9.1	4	10
Gravidity	1.4	1	11
Parity	0.4	0	7
Diabetes	No = 515, Yes = 37		
Birth weight (BW, g)	3401	1970	4750
Infant sex	Male = 286, Female = 266		
Delivery type	Vaginal = 506, Cesarean section = 46		

In this study, the umbilical artery pH value measured after delivery, an objective biochemical marker, was selected as the gold standard to separate the fetal state into normal and pathological classes. And the pH threshold was set to 7.15 after careful consideration [26]. A pH below 7.15 was agreed as pathological and a pH greater than or equal to 7.15 was classified as normal; thus, the database contained 447 normal and 105 abnormal FHR recording.

### Signal preprocessing

Preprocessing is an indispensable step in most biomedical signal processing applications and affect not only the values of extracted features but also the final classification performance. In clinical practice, the FHR signal has two typical acquisition methods: the CTG signal recorded externally by Doppler ultrasound (US) probe placed on the abdomen of pregnant women and the fetal electrocardiogram (FECG) signal measured internally by an electrode attached to the fetal scalp [9]. From this point of view, the FHR signal might be “contaminated” by noise due to many factors, such as the movement of mother and fetus, displacement of the transducer and external clinical environment.

**Fig. 2:**
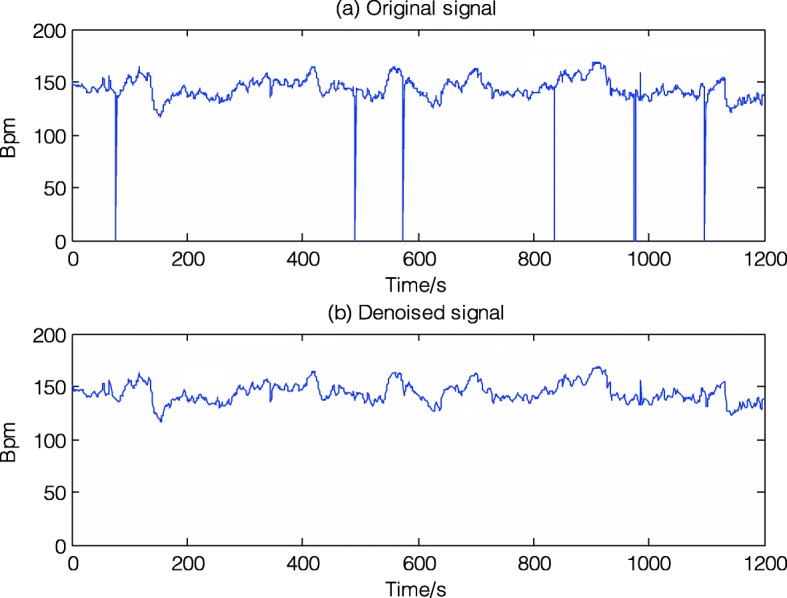
Signal preprocessing of No.1001 FHR recording (internal database number)

The noise of FHR signal usually manifests itself as artifact (or spiky) and missing (the period where the value of FHR is zeroed). Therefore, the primary goal of the preprocessing step is to reduce the two kinds of noise. First, a spline interpolation is used to fill the gap where the FHR value equals to 0 for no more than 15 s, otherwise long gaps (> 15 s) are removed directly. Second, a interpolation is again used between the first sample of the two adjacent points where the difference is higher than 25 bpm (beat per minute, the unit of FHR signal) and still the first of the new stable section which is defined as a time series of five adjacent samples with the differences among them less than 10 bpm. Finally, cubic spline interpolation is applied to replace the extreme (not physiological) values (< 50 bpm and > 200 bpm). Although the noise removal scheme is simple and more advanced techniques have recently been put forward, this preprocessing algorithm is effective and established necessary before any further analysis. Figure 2 shows the original noisy signal and preprocessed signal to be further analyzed (20mins in length).

### Continuous wavelet transform

Wavelet transform (WT), a widely used tool in the advanced field of signal processing, represents an effective method for multi-resolution analysis consisting of both time and frequency orientations [27]. As a type of WT, the CWT was originally proposed as an improved approach to address the issue of resolution [28]. The CWT has several outstanding merits, such as the reliable and flexible capacity to extract general and fine-grained feature information from the input signal; hence, this transform has been extensively employed in biomedical engineering to analyze non-stationary and nonlinear signals over the last decades [29]. The CWT is defined as the summation of the overall signal spectrum multiplied by the compressed and translated mother wavelet, which can be expressed mathematically by the following equations [23]:

1 $$ CWT\left(\tau, s\right)=\psi \left(\tau, s\right)={\int}_{-\infty}^{\infty }f(t){\varphi}_{\tau}^{\ast },f(t)\in {L}^2(R) $$

2 $$ {\varphi}_{\tau, s}(t)=\frac{1}{\sqrt{\mid s\mid }}\varphi \left(\frac{t-\tau }{s}\right),\tau, s\in R,s\ne 0 $$

3 $$ {\int}_{-\infty}^{\infty}\varphi (t) dt=0 $$

where, f(t) is input signal, φ(t) is wavelet basis, and ψ(τ,s) is wavelet coefficient, which is a function of two variables, τ and s, accounting for the translation and scaling factors, respectively. The former determines the degree to which the wavelet is compressed or stretched, while the latter reflects temporal and spatial information and represents the translation diameter of time shifting.

Instead of using traditional morphological analysis (baseline estimation, detection of acceleration and deceleration pattern, etc.), the primary reason for applying the CWT in this work is that the CWT provides a better way to observe and capture the local hidden characteristic information of the FHR signal in both the time and frequency domains simultaneously. Although the heart rate contained in a preprocessed FHR signal may not be estimated or lost in the time domain during the image transformation, Warmerdam et al. [30] still demonstrated that the CWT allowed clinicians to assess the reliability of spectral analysis of FHR recordings that were contaminated by artifacts: the mapping of the signals into a time-scale space and better visible localization of the frequency components in the analyzed signals.

In addition, Comert et al. also proposed a prognostic model using CWT to obtain 2D time-frequency image and achieved better performance in classifying the fetal state than time domain analysis [31]. However, they employed the conventional ML method: feature extraction (image-based time-frequency features, IBTF), feature selection (genetic algorithm, GA) and final classification (LS-SVM). Obviously, this approach was much more complex and obtained unsatisfactory result with the Se and Sp of 63.45 and 65.88%, respectively.

**Fig. 3:**
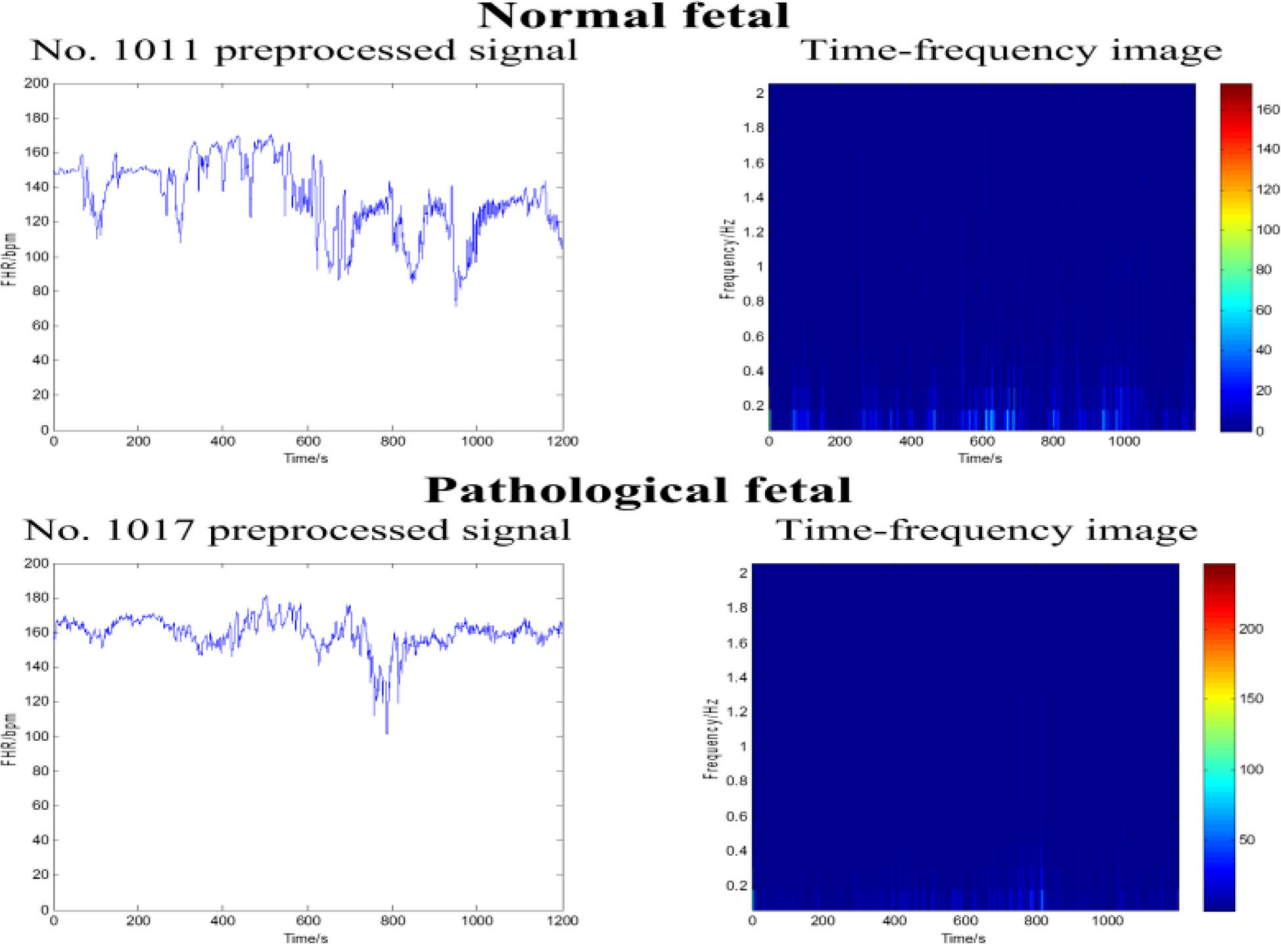
The FHR signals (left) and corresponding time-frequency images (right) of the normal (top) and pathological (bottom) classes using the CWT with the mother wavelet of db2 and a wavelet scale of 24

Figure 3 shows the preprocessed FHR signals and corresponding time-frequency images of a normal fetus and a pathological fetus using the CWT with the mother wavelet of db2 and a wavelet scale of 24. After careful consideration, two mother wavelets of db and sym with an order of 2 and three wavelet scales of 4, 5 and 6 were determined to enrich the database. Thus, the final dataset contained 3312 time-frequency images, including 2682 and 630 images for the normal (N) and pathological (P) fetal classes, respectively.

### Convolutional neural network

Serving as a typical type of DL, a CNN structure is composed of more hidden layers and neurons than the traditional multilayer perceptron (MLP) or ANN. Furthermore, the CNN algorithm is actually a type of supervised learning that can self-learn and self-organize based on the input data and corresponding output labels [18]. It eliminates the dependency on hand-crafted features and directly learns useful features from data. CNNs have already been successfully applied in many areas over the last decades, including face recognition, object localization, and image classification [19, 32, 33]. Due to the effectiveness of this approach, CNNs are extensively utilized in the medical field to design screening tools to assist clinicians [20–22].

The primary difference compared to traditional ML approaches is that a CNN can directly ignore the requirement for feature extraction and selection techniques. Hence, for most physiological signals, using CNNs can avoid the loss of valuable information and reduce the burden of computation in extracting and selecting the best features during the training process for accurate classification of pathological conditions. And a CNN significantly reduces the number of parameters that the neural networks need for training by means of receptive fields and weight sharing. The above attractive advantages were the main reasons why we chose a CNN for objective prediction of fetal acidemia.

**Fig. 4:**
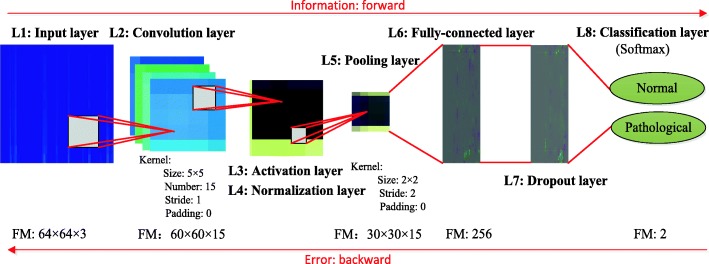
The CNN architecture proposed in this work. Note: L = layer; FM = output feature map or number of neurons (width ×height ×depth)

CNN is a combination of both feature extractor and classifier, and Fig. 4 illustrates the 8-layer deep 2D CNN architecture for this work consisting of the input layer, the convolution-activation-normalization-pooling layers, the fully-connected-dropout layers and the final classification layer. From input to output, the relationships between one layer and another layer are established through different computational neural nodes, and the input information is transferred layer by layer. The continuous convolution-pooling structure decodes, interprets, converges, and maps the characteristic information of the original data to the hidden feature space [34]. Next, a fully-connected layer executes the classification task according to the extracted features. The output shape gives the spatial size details of the output feature maps of each layer and the parameter represents the total number of weights including biases [35]. Detailed descriptions of the layers used in the CNN model are given below.

#### Image input layer (layer 1)

In this paper, the continuous wavelet transform is used to convert the original 1D time series into a 2D image as the input layer of the CNN. Simultaneously, in order to avoid overfitting, we applied the data augmentation technique of the CNN architecture in the input layer. A random crop method was employed for image transformation, which enriches the image dataset and improve the generalization ability of the model.

#### Convolution layer (layer 2)

A CNN is a form of deep neural network (DNN) with special convolution structure, which can reduce the amount of memory occupied by the deep network and the number of parameters in the network. In the convolution layer, a feature map in which hidden layers are connected to each other is used to extract pixel-level abstracted image features via convolution operations of one or more convolution kernels (also referred to as a filter) [36]. Each convolution kernel applies a sliding window mechanism to traverse the entire feature map, and thereby gathers and fuses the information of each small area to complete the representation of a partial feature of the input image. In a CNN, the filter parameters used in each convolution layer are ordinarily consistent for two reasons: (i.) sharing allows the image content to be unaffected by location; and (ii.) this consistency can dramatically reduce the optimization parameters. The mechanism of parameter sharing is a very important and attractive property of the CNN algorithm.

#### Activation layer (layer 3)

The result of the convolution layer is then mapped through an activation function (AF) to form the feature mapping relationship. The AF is generally used between the layers of a neural network [37], which performs a mapping transformation of the input data and provides the nonlinear modeling capability of the network. During the process, element-by-element calculations do not change the size of the original data. In this CNN model, the rectified linear unit (ReLU) is selected due to the following advantages compared to other linear functions: (i.) faster convergence speed; and (ii.) only one threshold is required to obtain the activation value without having to complete complex computations.

#### Normalization layer (layer 4)

The batch normalization (BN) layer is to standardize the input data of each layer during the training process of the neural network, so that the gradient becomes larger, avoiding the problem of gradient disappearance and greatly accelerating the training speed [18].

#### Pooling layer (layer 5)

In general, the CNN model inserts a pooling layer (also named a sub-sampling layer) periodically between consecutive convolution layers [18]. Since the image features that are useful in one region may be equally applicable in another area, the pooling layer incorporates semantically similar features. The pooling operation reduces the eigenvectors of the convolution output and the number of parameters, so pooling can lower the model complexity and speed up the computation while preventing overfitting. Similar to the convolution layer, the pooling operation performs feature mapping for each sub-region on the input feature map in steps of stride. Max pooling, average pooling and randomized pooling are the most common pooling methods. The former operation calculates the maximum value of the image area as the pooled result, which is used for this CNN model.

#### Fully-connected layer (layer 6)

The fully-connected layer is located at the end of the network structure and is a traditional MLP network [38]. The final output of this network layer is high-level features of the input images, which are then statistically calculated according to a classifier, and the probability of the corresponding class label for the input image is also computed. After several rounds of convolution and pooling processing, the input image information can be assumed to have been abstracted into more information-intensive features. The convolution layer and pooling layer can be considered the necessary approaches to automatic image feature extraction. And when the feature transformation is completed, the fully-connected layer is used to execute the final classification task.

#### Dropout layer (layer 7)

For classification, we usually attempt to avoid the occurrence of the overfitting, where the trained model obtains high accuracy on the training data, yet the generalization error on the test data is relatively large. In other words, overfitting refers to certain situation in which a defined model can memorize the random noise in the training data but is not able to learn the general trend of the training data. Many factors can lead to overfitting and the following specific solutions are available and proposed in this work [39]:

(a.) Regularization: Regularization is a powerful approach to solve an ill-posed problem to prevent overfitting by introducing additional information. L2 regularization is applied to add a regularizer to the cost function for this work.

(b.) Dropout technique: The dropout layer is usually arranged after the fully-connected layer. During the training process, several neural units are temporarily dropped from the network with a certain probability.

#### Classification layer (layer 8)

Finally, the classification layer is used to separate output classes using softmax function, namely, normal and pathological.

In our experiment, Table 2 presents the detailed parameters for each layer of the proposed CNN model, which were proved that there was not much effect on classification performance after careful observation.

**Table 2:** The detailed parameter settings for each layer of the proposed CNN model

Layer	Type	Parameter/Method	Value/Approach
1	Image input layer	Data augmentation	Random crop
		Data normalization	Zero center
2	Convolution layer	Stride	[1]
		Padding	0
		Learning rate of the weight	1
		Learning rate of the bias	1
		L2 regularization for the weight	1
		L2 regularization for the bias	1
3	Activation layer	Method	ReLU
4	Normalization layer	Alpha	1 × 10^−3^
		Beta	0.75
		K	2
5	Pooling Layer	Method	Max pooling
		Pool size	2 × 2
		Stride	[2]
		Padding	0
6	Fully-connected layer	Learning rate of the weight	1
		Learning rate of the bias	1
		L2 regularization for the weight	1
		L2 regularization for the bias	1
7	Dropout layer	Probability	0.5
8	Classification layer	Softmax	Cross-entropy

### Performance evaluation

To evaluate performance, we adopted the Se, Sp, and Acc indicators, which were calculated from the common binary confusion matrix. In addition, an imbalanced dataset (the ratio of positive to negative was approximately 4:1 in this work) can negatively affect the overall performance of any classifiers; thus, a quality index (QI) defined as the geometric mean of the Se and Sp, and the area under the receiver operating characteristic (ROC) curve (AUC) were also applied to alleviate this issue.

4 $$ Acc=\frac{TP+ TN}{TP+ FP+ FN+ TN} $$

5 $$ Se=\frac{TP}{TP+ FN} $$

6 $$ Sp=\frac{TN}{FP+ TN} $$

7 $$ QI=\sqrt{Se\cdot Sp} $$

where TP, FP, FN and TN represent true positive, false positive, false negative, and true negative respectively. In this work, the normal fetal state (N) is considered positive, and the pathological fetal state (P) is negative.

## Results

### Experimental setup

The current work designed a novel CAD system that combined the CWT and 2D CNN to assess fetal state. The proposed methodology was implemented in MATLAB 2017a (Natick, MA USA) software, and the CNN model was trained on a PC workstation with two Intel Core 3.70 GHz (i3–4710) processors and 4 GB of RAM.

In this study, ten-fold cross-validation was applied in the performance evaluation to obtain more reliable results. The total images were randomly separated into 10 segments and 90% (2414 N and 567 P) formed the training set while the remainder (10%, 268 N and 63 P) was used to test the performance of our proposed system. The process was repeated 10 times and the final results were averaged.

Then, the 2D images were considered as input for the CNN classifier. Figure 4 presents the structure of the designed CNN model consisting of 8 layers proposed in this paper. After careful experimentation, we set the hyperparameters of each layer and the training options as detailed in Tables 2 and 3, which did not have much effect on the classification performance.

**Table 3:** The detailed training settings of the proposed CNN model

Parameter		Value/Approach
Backpropagation algorithm		Stochastic gradient descent
Momentum		0.9
Initial learning rate		0.01
Learning rate drop	Factor	0.1
	Period	10 epochs
L2 regularizer factor		1 × 10–4

### Experiment one: optimization of the CNN parameters

**Fig. 5:**
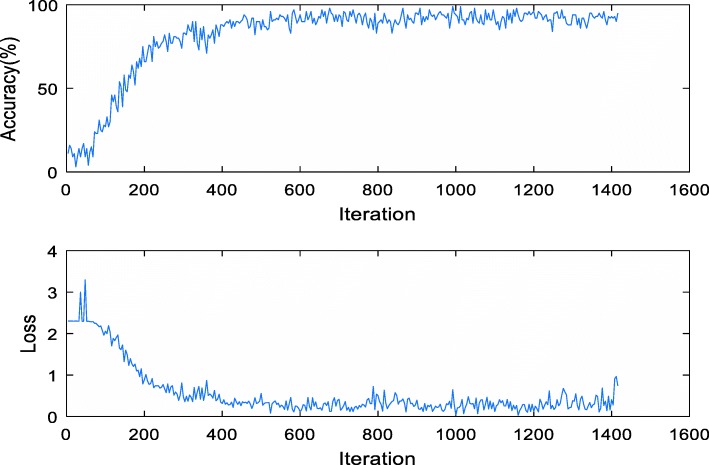
The training Acc (top) and loss (bottom) change with iteration during the CNN training process

When CNN training began, the internal status of the neural network was not visible to the researchers due to its black-box property. During the procedure, we required several indexes to observe and ascertain the training performance, such as the training loss and Acc. As shown in Fig. 5, the performance improved with the training iteration: Acc increased and loss decreased.

In CNN training, tuning the parameters is an indispensable step and plays an important role in optimization. After comprehensive experimentation, except the parameters defined in Tables 2 and 3, we discovered that the size of the convolution kernel, number of filters, maximum number of epochs, and size of the mini-batch could influence the classification performance. And the relevant experimental results can be summarized regarding the following two aspects.

**Fig. 6:**
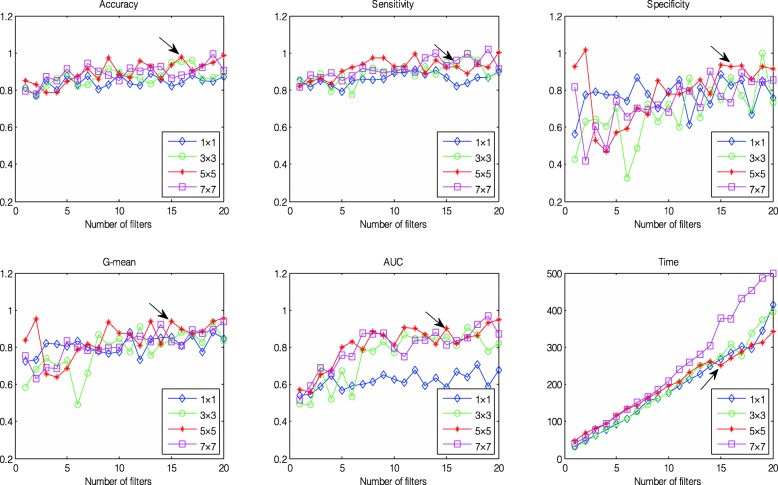
Comparison of the averaged classification performances using different kernel sizes and numbers of filters across ten folds. From left top to right top: Acc, Se, and Sp; from left bottom to right bottom: QI, AUC, and time

First, the parameters of the size of the convolution kernel (Para1) and the number of filters (Para2) greatly influenced image classification performance. For Layer 2, both of these parameters determined the size of the output feature map in width, height, and depth. Given Para3 = 20 and Para4 = 50, the effects of Para1 and Para2 were initially investigated. In the current work, Para1 was 1 × 1, 3 × 3, 5 × 5, and 7 × 7, and Para2 was in the range of 1 to 20 and was increased by 1, as demonstrated in Fig. 6. We could draw two conclusions based on observing the figure:

(a.) The relationship between six indicators and Para2 was generally positive, regardless of Para1, indicating that the performance improved with an increase in Para2 with a cost in computation time;

(b.) No clear relationship was found between the measurements and Para1, but we still discovered that Para1 = 5 × 5 performed better and the corresponding training time was relatively shorter than that for the other settings.

Hence, after careful observation, Para1 was set to 5 × 5, and Para2 was set to 15 (indicated by the black arrow in Fig. 6), which were selected for Layer 2.

**Fig. 7:**
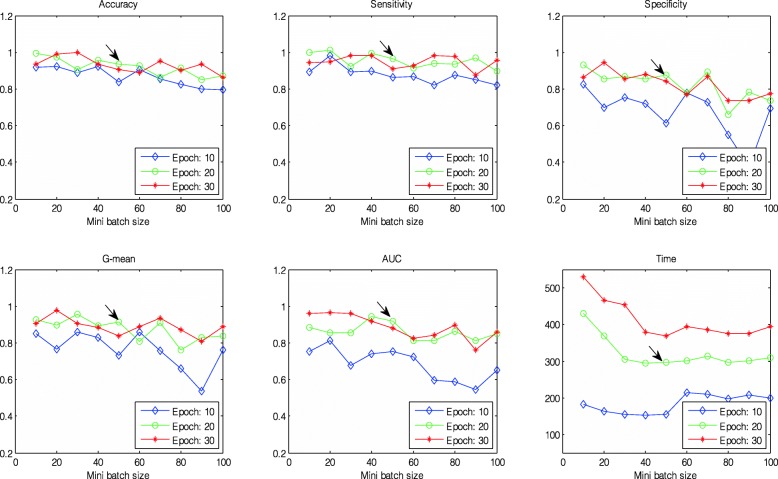
Comparison of the averaged classification performances using different max epochs and mini-batch sizes across ten-folds. From left top to right top: Acc, Se, and Sp; from left bottom to right bottom: QI, AUC, and time

Second, given Para1 = 5 × 5 and Para2 = 15, the training options of the CNN model were then experimented, including the maximum number of epochs (Para3) and the size of the mini-batch (Para4). These two parameters were known to have different degrees of influence on the performance of CNNs. In this paper, the values of Para3 and Para4 were in the ranges of 10 to 30 and 10 to 100, respectively, with both increased by 10, as depicted in Fig. 7. The following conclusions could be drawn from the figure:

(a.) The relationships between six indicators and Para4 were generally negative, regardless of Para3, signifying that the performance worsened and the training time was decreased with an increase in Para4;

(b.) The performance using Para3 = 20 was nearly similar to that with Para3 = 30 and better than that with Para3 = 10, but the training time for the former was much shorter than that for the latter.

Therefore, we determined that the optimum parameters (indicated by the black arrow in Fig. 7) were Para3 = 20 and Para4 = 50.

Finally, we also investigated the influence of difference layers. It can be observed from Table 4 that the relationship between the layers of CNN model and performance is not positive. Thus, we selected the 5-layer (i.e., 8-layer including the ReLU layer, normalization layer, and dropout layer) CNN architecture for higher Acc and less training time, as demonstrated in Fig. 4.

**Table 4:** Comparison of the averaged classification performances of different layers of CNN model across ten folds

Layers	Type	Performance					
		Acc (%)	Se (%)	Sp (%)	QI (%)	AUC (%)	Training Time (second)
**5**	**I – C – P – F – O**	**92.13**	**93.45**	**91.22**	**92.33**	**92.34**	**140.5**
6	I – C – P – C – F - O	91.88	92.55	89.74	91.13	91.15	162.3
7	I – C – P – C – P – F - O	91.21	92.13	89.25	90.68	90.69	178.8
8	I – C – P – C – P – F – F – O	90.76	91.71	88.67	90.18	90.19	201.3
9	I – C – P – C – P – C – F – F - O	91.34	92.34	89.56	90.94	90.95	225.4
10	I – C – P – C – P – C – P – F – F - O	90.82	91.88	89.11	90.48	90.50	248.2

Note: The best performance is indicated in bold. I = image input layer, C = convolution + ReLU + normalization layer, P = max pooling layer, F = fully-connected + dropout layer, O = classification output layer

### Experiment two: test of the CNN model

**Fig. 8:**
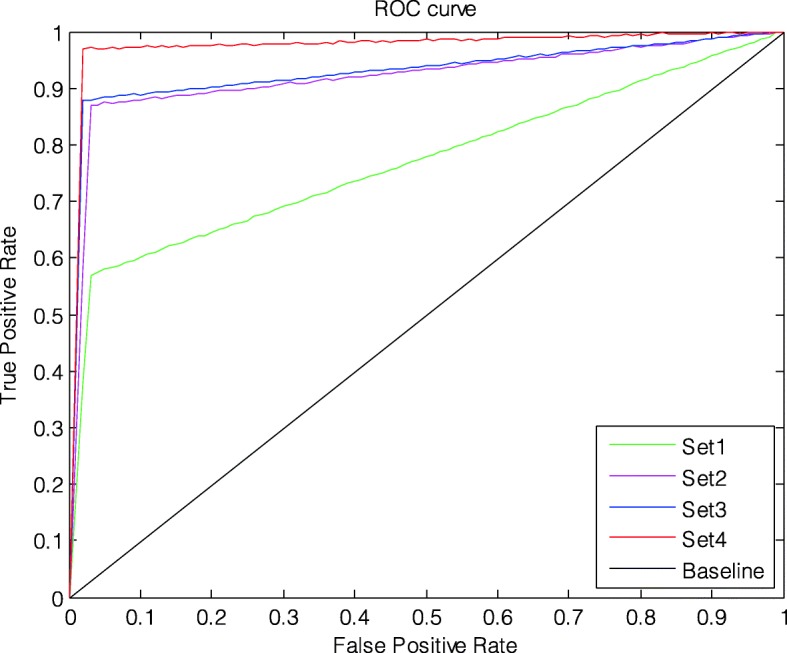
ROC curve of the proposed algorithm using different image resolutions and same optimization method

According to experiment one with the input image size of 28 × 28 × 3 RGB three channels, we confirmed four parameters of the 8-layer CNN model to achieve optimal performance: Para1 = 5 × 5, Para2 = 15, Para3 = 20 and Para4 = 50. We then tested the performance of our proposed algorithm using different image resolutions with the same optimization method. The original image size (420 × 560 × 3) was reduced to 16 × 16 × 3, 28 × 28 × 3, 36 × 36 × 3, and 64 × 64 × 3, which constituted four distinct datasets, denoted as Set1, Set2, Set3, and Set4. Table 5 clearly shows that with a higher resolution, the five measurements all increase significantly. The ROC curve presents in Fig. 8 further confirms this finding. In summary, when the size of the input images was 64 × 64 × 3, the proposed CNN model achieved the best classification performance (Acc = 98.34%, Se = 98.22%, Sp = 94.87%, QI = 96.53%, and AUC = 97.82%). Unfortunately, this performance increased came at a large cost in terms of computation power (Time = 1775s).

**Table 5:** Comparison of the averaged classification performances of different image resolutions using the same optimization method across ten folds

Measurement	Acc (%)	Se (%)	Sp (%)	QI (%)	AUC (%)	Time (second)
Dataset						
Set1	88.47	89.12	82.33	85.66	77.28	150
Set2	94.22	96.92	86.11	91.36	92.03	317
Set3	96.44	97.02	92.04	94.50	94.66	587
Set4	**98.34**	**98.22**	**94.87**	**96.53**	**97.82**	**1775**

Note: The best performance is indicated in bold

## Discussion

In this work, a novel CAD system based on the CWT and 2D CNN was proposed to assist obstetricians in making objective decisions regarding fetal status. We experimentally obtained better performance by tuning several parameters of the CNN model. According to Figs. 6 and 7 and Table 4, the optimal parameters were clearly fixed after full consideration. Furthermore, Table 5 shows that the overall classification performance improves with higher image resolution using the same training settings, yet the greatest disadvantage is that longer training times are required.

During the experiment, four different methods to obtain the 2D image as the input layer are tested in total, Table 6 gives a detail overview of performance. Unfortunately, we discovered that both Hilbert-Huang Transform (HHT) and Gabor Transformation could only achieved the accuracy below 80%. Although Short Term Fourier Transform(STFT) has achieved a relatively good result, its accuracy is still not good enough compared with CWT. According to our current research and analysis, it may be that CWT solves the resolution problem of STFT well and achieves multi-resolution feature analysis, which is more conducive to feature self-learning of CNN.

**Table 6:** Average classification performance for different input layers

Scheme	Performance (Validation)				
	Acc (%)	Se (%)	Sp(%)	QI(%)	AUC(%)
HHT	79.50	79.71	79.29	79.52	79.63
Gabor Transformation	76.38	80.56	72.33	76.25	77.22
STFT	83.27	86.78	78.83	82.91	83.10
CWT	98.34	98.22	94.87	96.53	97.82

Table 7 provides a summary of the different approaches proposed by researchers during the last decades for automated assessment of fetal well-being using FHR signals. Unfortunately, not all of these studies were performed using the same database (private or public); thus, comparisons among the studies are difficult. Nevertheless, Table 7 still demonstrates that the previous studies have all used identical strategies: signal preprocessing, feature extraction, feature selection and final classification. However, our proposed algorithm does not perform the feature extraction and selection stages; all the feature engineering steps are embedded in our CNN model for signal classification, representing the unique advantage of DL compared with conventional ML methods. Experiments using different means of feature extraction or selection are not required; in other words, we do not need to extract and select an optimum set of informative features. We can draw several conclusions from Table 7:

**Table 7:** Summary of related works conducted for the intelligent assessment of the fetal state using FHR signals obtained from CTG

Author	Database	Distribution (N/P)	Method			Performance(%)
			Feature extraction	Feature selection	Classifier	
Krupa et al. 2011 [13]	Private	30/60	EMD	/	SVM	Acc:87 Se:95 Sp:70
Spilka et al.2012 [12]	Private	123/94	33 Set1, Set2, Set3	PCA,IG	NB,SVM,DT	Se:73.4 Sp:76.3 Fm:71.5
Czabanski et al. 2012 [14]	Private	146/43	7 Set1	/	WFS+ LS-SVM	Acc:92.0 QI:88.2
Fanelli et al. 2013 [15]	Private	61/61	2 Set3	/	ST	AUC:75
Xu et al. 2014 [40]	Private	255/255	64 Set1, Set2, Set3	GA	SVM	Se:83 Sp:66 AUC:74
Dash et al. 2014 [41]	Private	60/23	8 Set1	/	GM,NB	Se: 61 Sp:82
Spilka et al. 2014 [42]	CTU-UHB	175/377	33 Set1,Set2, Set3	/	LCA + RF	Se:72 Sp:78
Doret et al. 2015 [11]	Private	30/15	12 Set2, Set3	/	ST	AUC:87
Comert et al. 2016 [43]	CTU-UHB	60/40	18 Set1, Set2	/	ANN	Acc: 87.0 Se:88.7 Sp:85.1
Stylios et al. 2016 [44]	CTU-UHB	508/44	54 Set1, Set2, Set3	AUC	LS-SVM	Se:68.5 Sp:77.7
Comert et al. 2016 [16]	CTU-UHB	272/280	11 Set2, Set3	/	ANN	Acc: 92.40 Se:95.89 Sp:74.75
Georgoulas et al. 2017 [45]	CTU-UHB	508/44	33 Set1, Set2, Set3	AUC	LS-SVM	Se:72.12 Sp:65.30
Comert et al. 2018 [31]	CTU-UHB	439/113	IBTF	GA/	LS-SVM	Se:63.45 Sp:65.88
Li et al. 2018 [21]	Private	3012/1461	FHR + 1D CNN			Acc:93.24
Comert et al. 2018 [22]	CTU-UHB	508/44	STFT+2D CNN			Se:56.15 Sp:96.51 QI:73.61
**Current work**	**CTU-UHB**	**447/105**	**CWT + 2D CNN**			**Acc:98.34** **Se:98.22** **Sp:94.87** **QI:96.53** **AUC:97.82**

Note: The best performance is indicated in bold

(a.) Compared with [30], based on the same database (CTU-UHB) and image transformation method (CWT), our approach performs much better (Se = 98.22 and 63.45%, Sp = 94.87 and 65.88%), which further highlights the superiority of CNN over ML.

(b.) Compared with [20], although the test database is different, the 2D CNN model obtains higher accuracy than 1D CNN (Acc = 98.34% and 93.24).

(c.) Compared with [21], based on the same database and 2D CNN model, the CWT can better reflect the characteristic information of FHR signal than STFT according to the time-frequency image (Se = 98.22 and 56.15%, Sp = 94.87 and 96.51%, QI = 96.53 and 73.61%).

(d.) To the best of our knowledge, this CNN algorithm achieved better classification performance in predicting fetal state using FHR signals compared with other related works, as presented in Table 7.

In summary, the proposed system has several attractive advantages: (i.) feature extraction and selection techniques are not required; (ii.) the CWT is used to obtain 2D time-frequency images, which is believed to reflect the hidden characteristics of the FHR signals in both the time and frequency domains; (iii.) an 8-layer deep 2D CNN is implemented and its parameters are tuned to obtain better performance; and (iv.) this approach performs best among the state-of-the-art methods.

Nevertheless, the proposed system has some drawbacks: (i.) the training of the CNN model requires a very large amount of diverse data; and (ii.) the algorithm is computationally intensive in learning useful features from the input images.

In fact, if this classification algorithm can accurately discriminate between normal and pathological classes, then the long training time will be secondary in medical fields. Once the CAD system designed by the proposed algorithm is successfully trained, the system can immediately distinguish an unknown fetal state. Fortunately, some solutions are available to overcome the drawbacks of our proposed system. We can enrich the dataset using image transformation, such as rotation, cropping and whitening, etc. Then, training CNN models integrated with a graphics processing unit (GPU) will help significantly decrease training time and power consumption since one of the important properties of the CNN algorithm is its concurrency.

## Conclusions

The accurate diagnosis of fetal acidemia caused by hypoxia can allow obstetricians to intervene in a timely manner and take appropriate action to prevent permanent damage to the fetus. In clinical practice, the FHR signal is a commonly used tool to monitor the fetal state during labor and delivery. However, a visual analysis of the FHR signal with the naked eye is a challenging task for obstetricians since this type of assessment is subjective and irreproducible. Visual interpretation easily leads to significant inter-observer and intra-observer variability. Therefore, implementing a CAD system in clinical settings will guarantee the rapid and accurate prediction of fetal distress more objectively.

In this study, our primary contribution is to propose a data-driven approach to automatically assess the fetal state using a deep CNN. After signal peprocessing, the input time-frequency images were obtained using the CWT with different types of mother wavelets and wavelet scales. After comprehensive experimentation focused on tuning the parameters and changing the image sizes, we achieved the best classification performance with the optimum configuration (8 layers, size of the convolution kernel = 5 × 5, number of filters = 15, maximum number of epochs = 20, size of the mini-batch = 50, and image resolution = 64 × 64 × 3), and the averaged Acc, Se, and Sp were 98.34, 98.22, and 94.87% across ten folds, respectively. To alleviate the influence of the class imbalance phenomenon, QI and AUC indicators were also applied to measure the overall performance with values of 96.53 and 97.82%, respectively. Since using features is susceptible to bias in extracting (selecting) the features and limits the ability of a classifier to fully learn from the data, the CNN-based framework obviated the requirement for feature engineering (i.e., feature extraction and selection). Overall, the results proved the effectiveness of our proposed CAD system, which can be introduced into clinical practice and assist obstetricians in making accurate medical decisions objectively.

The results are promising and provide the baseline for future research involving strategies without feature extraction and selection and entirely relying on the neural network model for fetal state assessment. GPUs will be integrated into the workstation to reduce the complexity and speed up the training process in terms of computation. In addition, we will combine FHR signal with other biomedical signals (e.g., UC) to improve the accuracy for providing more reliable decision tool. To make the system more explainable for the obstetricians and pregnant women is also a huge challenge.

## Data Availability

The data used in this work is publicly available from http://www.physionet.org/physiobank/database/ctu-uhb-ctgdb/.

## Abbreviations

Acc: Accuracy
AI: Artificial Intelligence
ANN: Artificial Neural Network
AUC: Area under the ROC curve
CAD: Computer Aided Diagnosis
CNN: Convolutional neural network
CWT: Continuous Wavelet Transform
DL: Deep learning
DT: Decision Tree (C4.5)
EMD: Empirical Mode Decomposition
FHR: Fetal Heart Rate
Fm: F-measure
FN: False Negative
FP: False Positive
GA: Genetic Algorithm
GE: Grammatical Evolution
GM: Generative Model
IBTF: Image-based Time-frequency
IG: Information Gain
LCA: Latent Class Analysis
LS-SVM: Least Square SVM
ML: Machine Learning
N: Normal
NB: Naive Bayes
P: Pathological
PCA: Principle Component Analysis
QI: quality index
RF: Random Forest
Se: Sensitivity
Set1: Morphological
Set2: Linear
Set3: Nonlinear
SMOTE: Synthetic Minority Oversampling Technique
Sp: Specificity
S-SVM: Sparse SVM
ST: Statistical Test (*p*-value)
STFT: Short Term Fourier Transform
SVM: Support Vector Machine
TN: True Negative
TP: True Positive
WFS: Weighted Fuzzy Scoring
